# Who or What Influences the Individuals’ Decision-Making Process Regarding Vaccinations?

**DOI:** 10.3390/ijerph17124461

**Published:** 2020-06-21

**Authors:** Hanna Czajka, Szymon Czajka, Paweł Biłas, Paulina Pałka, Szczepan Jędrusik, Anna Czapkiewicz

**Affiliations:** 1 Faculty of Medicine, University of Rzeszów, 35-959 Rzeszów, Poland; hanna.czajka@onet.pl; 2 St. Louis Regional Specialised Children’s Hospital, 31-503 Krakow, Poland; 3 Department of Anesthesiology and Intensive Care, School of Medicine in Katowice, Medical University of Silesia in Katowice, 40-752 Katowice, Poland; 4 Student’s Scientific Society, Faculty of Medicine, University of Rzeszów, 35-959 Rzeszów, Poland; pawelbilas94@gmail.com (P.B.); paula.p986@gmail.com (P.P.); jedrusik.szczepan@gmail.com (S.J.); 5 Faculty of Management, AGH University of Science and Technology, 30-067 Kraków, Poland; gzrembie@cyf-kr.edu.pl

**Keywords:** vaccination, vaccine hesitancy, vaccine-preventable diseases, antivaccine movements, medical education

## Abstract

Thanks to vaccines, many people are not exposed to the risks associated with vaccine-preventable diseases (VPDs). This, however, results in growing popularity of antivaccine movements and affects global and local epidemiological situation. Vaccine hesitancy has become a significant problem not only for epidemiologists but also for practitioners. Fortunately, the hesitant group seems to be vulnerable to intervention, and studies indicate that these patients can be persuaded to undergo vaccinations. The aim of the present study was to determine the factors most strongly affecting vaccination-related attitudes and decisions. An anonymous, self-administered survey consisting of demographic data and single select multiple-choice questions regarding vaccination was conducted. The voluntary study included secondary school pupils, medical and nonmedical students, healthcare professionals, hospital and clinic patients as well as parents. A total of 7950 survey forms were distributed between January 2018 and June 2019 in south-eastern Poland. A total of 6432 respondents (80.2%) completed a questionnaire that was eligible for analysis. The positive attitude toward vaccination was significantly affected by older age, by the fact of obtaining information on vaccinations from a physician, this information’s higher quality (assessed in school grade scale), higher level of knowledge on vaccines and by the fact of denying the association between vaccination and autism in children (*p* < 0.001). The probability of supporting vaccinations was almost eight-fold lower among respondents believing the vaccine–autism relationship. Chance of supporting vaccination doubled in the group with a higher knowledge level. The individuals not provided with expert information on vaccination were twice as often unconvinced. Age, education and having children significantly affected the attitude toward influenza immunization (*p* < 0.001). Older, better educated respondents and those having children were more positive about vaccinations. The medical community still exert decisive effects on attitudes toward vaccinations. High-quality information provided by them is of great importance. Skillful and competent provision of evidence-based information disproving the myth about vaccine–autism connection and proper education of medical staff is essential in molding positive attitudes toward vaccinations.

## 1. Introduction

Over the course of two centuries, widespread vaccinations have contributed to the global eradication of smallpox, elimination of poliomyelitis in the majority of countries and a significant decline in overall morbidity and mortality caused by infectious diseases. This accomplishment was placed at the very top of the list of public health achievements of the 20th century published by the Centers for Disease Control and Prevention (CDC) [1].

Paradoxically, vaccines have become a victim of their own success. Due to the popularity and high effectiveness of this form of prevention, many people are not exposed to the risks associated with vaccine-preventable diseases (VPDs). Hence, there are increasingly common opinions of patients and their parents on limited benefits versus high risks of mass and routine vaccination programs [2]. For almost two decades, the growing issues of avoiding preventive vaccination and concerns about the safety and effectiveness of vaccines have been observed and constantly fomented by activists of antivaccination movements [3,4]. With widespread and unlimited access to various, often unreliable sources of information, antivaccine movements supported by effective propaganda have become an international problem with a global reach [5].

Concerns about vaccination have become a global phenomenon and led to the search for answers to the question, who has the greatest influence on the parental attitude toward vaccination—medical professionals, internet or family and social environment [6]. In recent years, the role of Facebook as a source of pro and antivaccine information has also been analyzed [7]. Another research on parents’ attitudes toward vaccinating their children includes new methods of data collection via smartphones, which gives quick results with low data acquisition costs compared to traditional survey methods [8]. Public health authorities are encouraged to use such tools in order to improve the collection of data on the public perception of prevention of infectious diseases.

In 2012, the World Health Organization (WHO) Strategic Advisory Group of Experts on Immunization (SAGE) created the Working Group on Vaccine Hesitancy [9]. The purpose of this group is to recognize the reasons for vaccine hesitancy to assess why children and adults are under-vaccinated or unvaccinated. Vaccine hesitancy results in the delay in the realization of vaccination schedules, also in countries where full availability of this form of prophylaxis is ensured [10]. Research indicates that the level of knowledge of health professionals carrying out vaccinations (doctors and nurses) and their personal attitudes toward vaccination is important in increasing immunization rates [11]. According to the Working Group definition “vaccine hesitancy refers to delay in acceptance or refusal of vaccination despite availability of vaccination services.” The most vulnerable to external influences during their decision-making process are the patients and their caregivers who hesitate rather than those who are declared opponents [12]. In a Canadian survey, only 3% of parents categorically refused all vaccines for their children, while 19% considered themselves to be vaccine hesitant [13]. Health professionals have therefore the opportunity to convince undecided individuals of the importance, high effectiveness and safety of preventive vaccinations, thereby contributing to the improvement of local and global epidemiological situations.

One of the most frequently attacked vaccines is the measles, mumps and rubella vaccine (MMR) administered in the pediatric population. However, the theories disseminated by opponents of vaccination also result in a decrease in confidence in this form of infectious disease prevention among adults. A consequence of the decline in public confidence in the MMR vaccine is the increased number of measles cases recorded in recent years in both the United States and Europe [14,15].

The Polish Vaccination Programme is a mixed system composed of mandatory and recommended vaccinations. Compulsory vaccinations for children, adolescents and high risk groups include immunization of infants against tuberculosis (BCG) and viral hepatitis (HBV) at birth, and in the following months vaccinations against: tetanus, diphtheria and pertussis (DTP), Hemophilus Influenzae type b, Streptococcus pneumoniae, Poliomyelitis and against measles, mumps and rubella (MMR). Vaccination against varicella is carried out free of charge only in high-risk groups, and vaccinations against rotaviruses, Neisseria meningitidis type B and ACWY, human papillomavirus (HPV) and hepatitis A (HAV) are recommended but performed privately, without the financial support of the state. In our schedule, whole-cell pertussis vaccines have been used for years, and 5- or 6-in-1 vaccines can also be used alternatively at the expense of the child’s parents. The list of obligatory and recommended vaccinations, as well as details related to the realization of the vaccination program, are published annually by the Chief Sanitary Inspector [16,17].

In Poland, the number of patients avoiding preventive vaccination is increasingly high, which is consistent with the global trends. According to the Polish Chief Sanitary Inspector, in 2018, about 43,000 individuals avoided compulsory vaccinations [18].

Considering the above data, the aim of the present study was to determine the factors most strongly affecting vaccination-related decisions in the Polish population. This study focuses on the social groups most closely involved in the decision-making process concerning vaccinations—health care workers, medical students, parents of patients and potential future parents.

## 2. Materials and Methods

### 2.1. Study Design and Self-Administered Questionnaire

The survey was conducted using an anonymous, self-administered questionnaire consisting of questions about demographic data and single select multiple-choice questions regarding vaccination (Table 1). The original questionnaire was developed by the researchers and passed the evaluation carried out by a sociologist expert in the field of vaccine hesitancy. The survey was conducted in person, one of the researchers was always present when the respondents filled in the paper version of the questionnaire, explaining any doubts if necessary. Each survey form contained a short explanation of the purpose of the study and instructions for completing the questionnaire. Before the survey was conducted, a pilot test was conducted with 50 participants who assessed the content of the questions and their comprehensibility. Comments from the participants of the pretest were used for adjusting the wording of some questions and the instructions for the respondents. The voluntary study included secondary school pupils, medical and non-medical students, healthcare professionals, hospital and clinic patients as well as parents of minor patients. These groups have been selected because it is these people who have the greatest influence on the decision-making process concerning vaccinations, and at the same time they are or will be the future parents of the underage patients. Depending on the problem raised, only those surveys in which the respondent provided the answer to the problem discussed were analyzed. Vaccination-related opinions of patients or their parents, secondary school pupils, students and healthcare professionals will be analyzed in three separate reports. The present paper analyzes the results of the entire study population.

### 2.2. Setting

The survey was conducted between January 2018 and June 2019 in randomly selected schools, universities, hospitals, primary care clinics and workplaces predominantly in south-eastern Poland. A total of 7950 questionnaire forms were distributed and the survey was carried out after obtaining the consent of the respective authorities (directors, the management of the establishments, university authorities). The study design was approved by the Bioethical Committee of the University of Rzeszow (reference number: 10/12/2018).

The analysis included the following demographic data: gender, age (four categories: 18–25, 25–35, 36–45 and >45 years), education (elementary school—8/9 years, secondary school—12 years, higher—university degree), financial status (bad, average, good) and having children or otherwise.

The attitudes of the respondents to vaccination was assessed on the basis of the answers to question B8:

- “Do you consider yourself an advocate or opponent of vaccination?”

The following multiple-choice questions were asked, specifying the respondent’s actions in relation to the declared attitude:

- “Have you been vaccinated within the last three years?”
- “Are you vaccinated against seasonal influenza?”

In addition to demographic variables, the following items concerning the main determinants of respondents’ attitudes toward vaccination were included:

- “Please indicate one source of information about vaccinations that you find most convincing” (1)-medical sources (doctor, nurse, pharmacist) (2)-media (press, radio, television, Internet) (3)-environment (family, friends)
- “Did you obtain information on vaccination from healthcare professionals during visits to primary care physicians (PCP) or specialist outpatient clinic?”: (1)-Yes, (2)-no.
- “Using the school grade system, assess the level of vaccination information provided by health professionals” (1 worst–6 best)
- “What was the most important source of negative opinions about vaccinations?”: (1)-medical sources, (2)-media, (3)-environment
- “What opinion about vaccination was provided by health professionals?”: (1)-fully positive, (2)-questioning the validity of certain vaccinations, (3)-strongly negating the purposefulness of vaccinations
- The following questions were used to assess the level of knowledge of respondents about vaccinations:
- “Which of the infectious diseases has been completely eliminated by vaccination?” (1)-measles, (2)-rabies, (3)-smallpox, (4)-whooping cough, (5)-hepatitis C
- “The statement that “vaccines cause autism” is:” (1)-true, (2)-false

### 2.3. Statistical Analysis

All collected data were entered into a database prepared in Excel 16.0. Statistical analysis was conducted using Statistica 13.1 (StatSoft, Inc., Tulsa, OK, USA). Summary statistics were presented (mean and standard deviation, median and quartiles, or absolute values and percentages, as appropriate). The answers to the questions asked in the survey were ranked. Ranks corresponding to particular answers are presented in Table 1. The U-Mann-Whitney or Kruskal-Wallis test was used to assess quantitative variables in two or more groups respectively. χ^2^ or exact Fisher’s tests were performed for independent qualitative variables. In this study logistic regression was applied. For the purposes of the logistic regression model, questions with answers represented by dichotomous variables were taken into account. The model was developed for questions B8 and B12. Classification (decision) trees were constructed. The statistical significance level was set to 5% (α = 0.05).

## 3. Results

### 3.1. Sociodemographic Data

Out of 7950 questionnaires distributed, 6432 completed and analyzable forms were returned (the response rate = 80.2%). The demographic characteristics of the study group are shown in Table 2. In the study population, 4483 (70.1%) (95%CI: 68.96–71.20) respondents were supporters of vaccination, only 193 (3%) (95%CI: 2.59–3.43) were strong opponents, while 26.8% (*n* = 1721) (95%CI: 25.82–27.99) were undecided and 35 (0.5%) did not respond to the question about vaccination-related attitudes. Since the number of opponents of vaccination was low, only vaccination advocates and undecided respondents were included in the analysis. The study population was therefore divided into two subgroups—convinced of vaccination and undecided. The between-group differences in gender, age, education, financial status and having children or otherwise were found to be statistically significantly different (*p* < 0.001).

### 3.2. Analysis of the Answers to the Question: “Do You Consider Yourself An Advocate or Opponent of Vaccination?”

**Figure 1:**
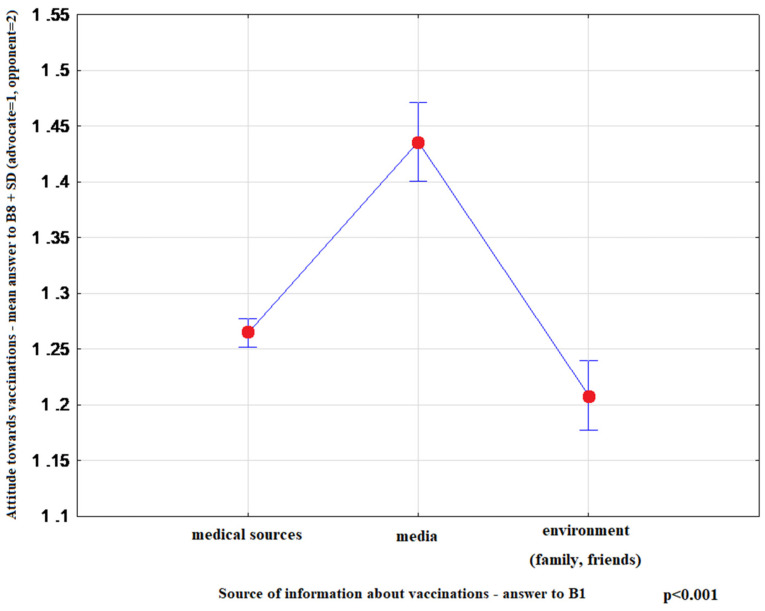
Mean rank-answer to the question about the attitude toward vaccination (B8) + SD depending on the preferred source of information on vaccination (B1). (medical sources *n* = 4718; media *n* = 668; environment *n* = 818).

**Figure 2:**
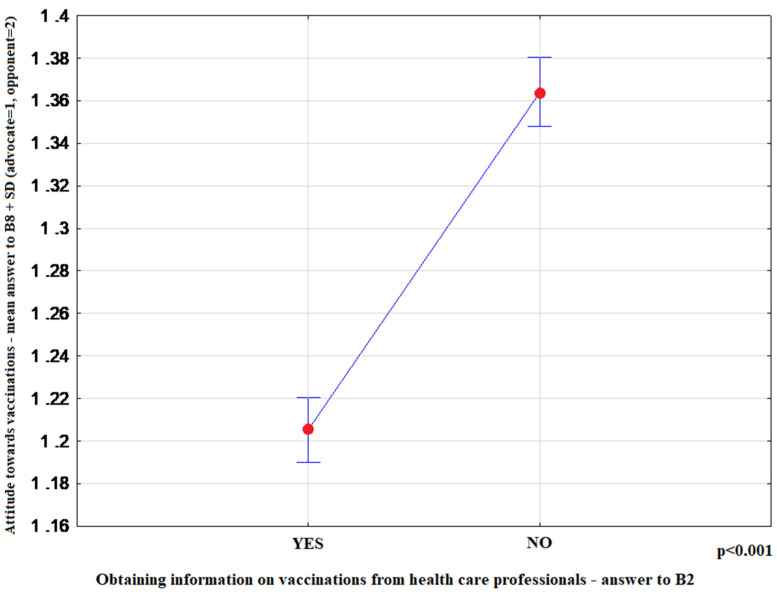
Mean rank-answer to the question about the attitude toward vaccination (B8) + SD depending on the fact of obtaining or not obtaining information on vaccinations from health care professionals (B2). (Yes *n* = 3365; No *n* = 2997).

**Figure 3:**
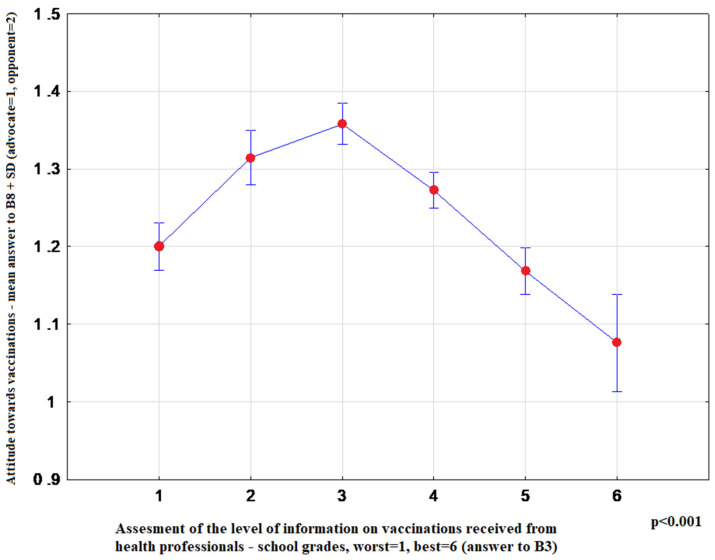
OY axis: Mean rank-answer to the question about the attitude toward vaccination (B8) + SD (advocate = 1; opponent = 2), OX axis: Level of information on vaccination received from health professionals-answers to B3-school grades system worst = 1, best = 6 (1 *n* = 820; 2 *n* = 614; 3 *n* = 1043; 4 *n* = 1422; 5 *n* = 794; 6 *n* = 194).

**Figure 4:**
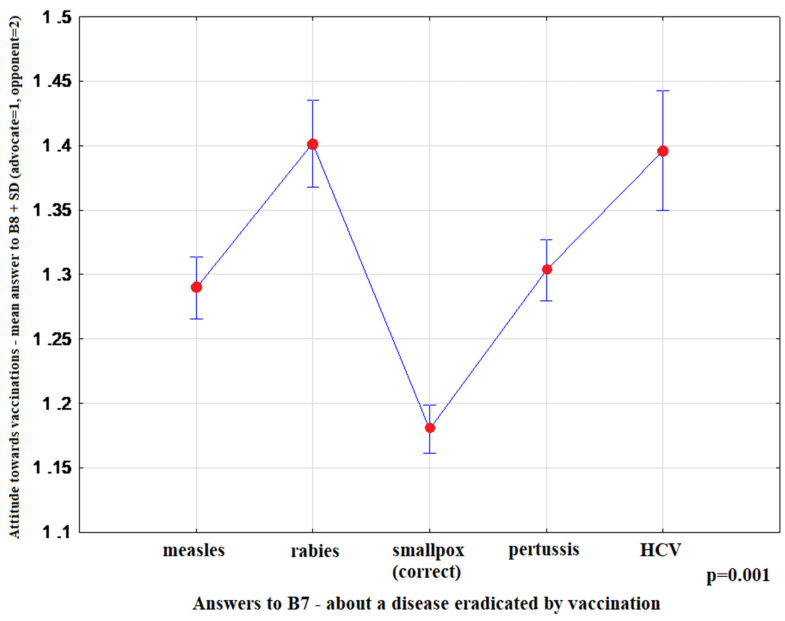
OY axis: Mean rank-answer to the question about the attitude toward vaccination (B8) + SD (advocate = 1; opponent = 2) OX axis: Answers to B7-about a disease eradicated by vaccination (measles *n* = 1358; rabies *n* = 695; smallpox-correct *n* = 2199; pertussis *n* = 1373; HCV (Hepatitis C Virus) *n* = 358).

**Figure 5:**
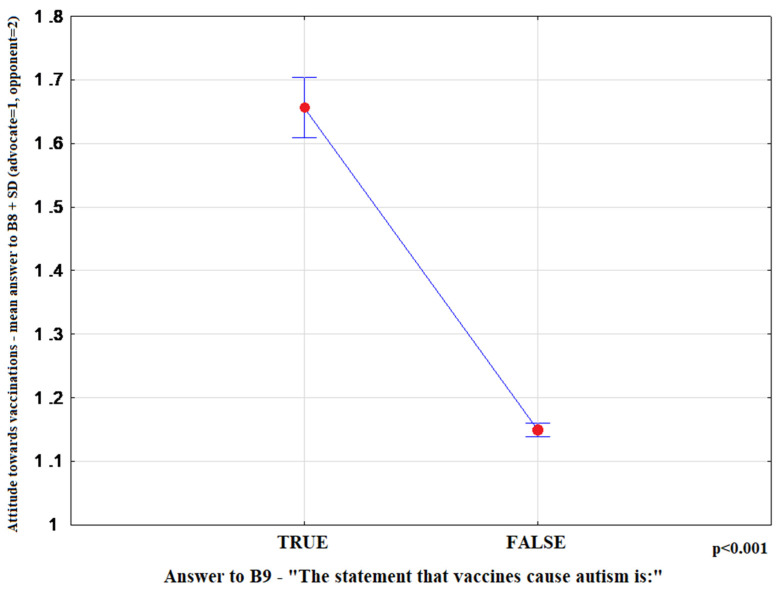
Mean rank-answer to the question about the attitude toward vaccination (B8) + SD depending on the opinion about the relationship between vaccines and autism (B9). Answers to B9: TRUE *n* = 299; FALSE *n* = 4410.

The most convincing sources of vaccination-related information chosen by respondents were as follows: health professionals-15.10% (95%CI: 14.20–16.00), media-65.81% (95%CI: 64.61–66.99), family and friends-19.09% (95%CI: 18.11–20.08). Media information on vaccination favoured indecisive attitudes toward this form of prevention (*p* < 0.001) (Figure 1). Moreover, advocates of vaccination more commonly indicated health professionals as their source of information (*p* < 0.001) (Figure 2), and more highly assessed the quality of their information (*p* < 0.001) (Figure 3). The information provided by family and friends had the most negative impact on attitudes toward vaccination (Figure 1). The incidence of correct answers to the question about diseases eradicated by vaccination (smallpox) was higher among vaccination advocates (*p* = 0.001) (Figure 4); a higher percentage of undecided respondents associated vaccinations with autism in children (*p* < 0.001) (Figure 5).

**Figure 6:**
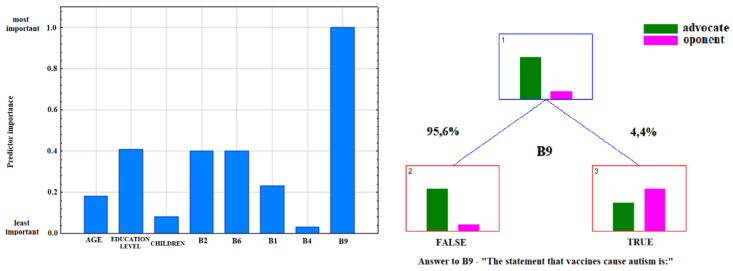
Ranking of predictors and decision tree for answer to the question B8: “Do you consider yourself an advocate or opponent of vaccination?” including question B9.

**Figure 7:**
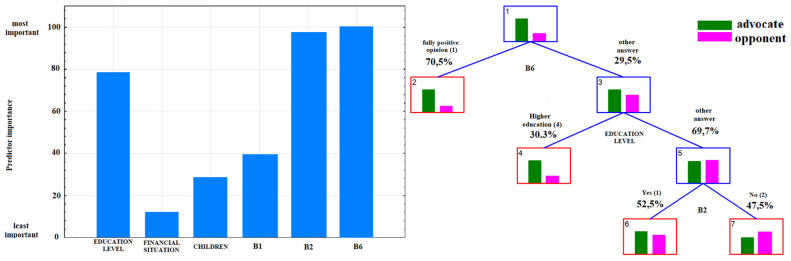
Ranking of predictors and decision tree for answer to the question B8: “Do you consider yourself an advocate or opponent of vaccination?” after excluding question B9.

According to the data presented in Table 3, the attitude toward vaccination was most significantly affected by age, the information provided by physicians, the quality of this information, knowledge of vaccine-preventable diseases and the fact of associating vaccination with autism in children (*p* < 0.001). Considering the odds ratios, the probability of supporting vaccinations was almost eight-fold lower among respondents believing that vaccinations caused autism (which was also confirmed by the ranking of predictors of attitudes toward vaccination, Figure 6). Other important factors influencing attitudes toward vaccination were the fact of receiving information on vaccination from health care workers (B2), the opinion expressed by these professionals (B6) and the level of education of the respondents (Figure 7). Among respondents who answered correctly the question about eradication of smallpox, the probability of being a supporter of vaccination doubled. The individuals who were not provided with expert information on vaccination twice as often declared themselves unconvinced. In adulthood (>45 years), the probability of a positive attitude toward vaccination was three times higher compared to the other age groups.

### 3.3. Analysis of the Answer to the Question: “Are you Vaccinated Against Seasonal Influenza?”

**Figure 8:**
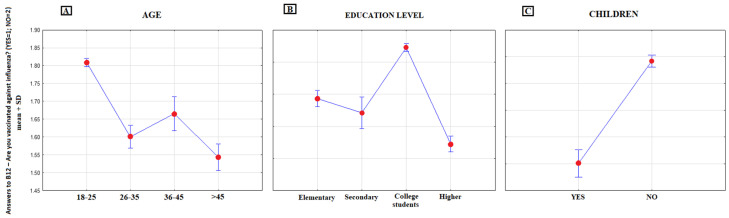
Mean rank-answers to B12: “Have you been vaccinated against influenza?” (YES = 1, NO = 2) in different demographical categories. (**A**) age groups, (**B**) education level groups, (**C**) groups on the basis of the fact of having children

In the study population, 424 (6.6%) respondents were annually vaccinated against influenza during the period of the last three years (95%CI: 6.40–7.70), 1104 (17.2%) were vaccinated at least one time (95%CI: 16.38–19.33) and 4875 (75.8%) (95%CI: 74.07–76.05) were never vaccinated against influenza; 29 (0.4%) respondents did not answer this question. The analysis of demographic data demonstrated that age, education and having children (*p* < 0.000) significantly affected the answers to this question. The number of individuals receiving vaccinations against influenza increased with age and was also found to be higher among those with children (Figure 8). According to Figure 8B, the percentage of individuals vaccinated against influenza decreased with education. Further analysis revealed that those responsible for the above result were students, as the majority of them were not vaccinated against influenza (85%—never vaccinated, 3.1%—vaccinated each year). Once this group of respondents was excluded, the result changed. Therefore, it can be concluded that the popularity of influenza vaccination increases with the level of education; after the exclusion of students, the education level is no longer statistically significant, as compared to other variables (Table 4).

## 4. Discussion

### 4.1. Advocates and Potential Opponents of Vaccination in the Study Population

The answers to the question “Do you consider yourself an advocate or opponent of vaccination?” demonstrate that the number of opponents is comparable to the estimates from other countries. According to the American data, the number of people refusing all types of vaccinations is < 2% (4, 24), which is a socially marginal group. Therefore, it is essential for physicians to focus on those unconvinced about the benefits of vaccinations. The above has also been strongly emphasized by the experts of the WHO SAGE Vaccine Hesitancy Working Group; undecided individuals can be convinced while declared opponents of vaccinations are resistant to substantive arguments [9,12].

According to the data published by the Centre for Public Opinion Research (in Polish, CBOS), negative attitudes toward vaccination are favored by young age, lower education and lower financial status [19]. The findings of our study conducted later confirm the CBOS observations. Otherwise, the data reported in the Italian study of 2017 differ from the above -mentioned results and demonstrate that younger parents with lower levels of education are more eager to have their children vaccinated [20]. In some other studies, less affluent respondents have been found to be more skeptical of vaccinations [21,22,23], which is consistent with our findings. It can therefore be assumed that demographic variables have different effects on attitudes toward vaccination and the results of various studies are inconsistent, depending on study locations and periods.

Furthermore, the vast majority of studies point to the leading role of health professionals in shaping social attitudes toward vaccination, especially specialists in infectious diseases and in internal diseases, pediatricians, family doctors, nurses and pharmacists. Our results demonstrate that both the provision of information by health professionals and the quality of their information are essential for the decision to vaccinate or otherwise (Figure 2 and Figure 3). Furthermore, the lack of vaccination information or poor quality of information result in reluctant attitudes to vaccination (Table 3). According to the review article by Shen and Dubey, the parents who are hesitant about vaccinations far outnumber strong opponents of vaccination. The authors conclude that family physicians, as a trusted source of information, play a key role in driving vaccine acceptance [24]. The Australian study highlights the importance of transparent, reliable and comprehensive information about vaccinations, vaccine-preventable diseases and possible adverse side effects of vaccines [25]. In our material, respondents who were not informed about vaccinations in primary care or specialist outpatient clinics were twice as likely to be distrustful of vaccinations (Table 3).

Health professionals who provide vaccination-associated information should be alarmed by the conviction/fear of patients and their care givers that vaccines can induce autism. Our results suggest that such a conviction significantly affects negative attitudes toward vaccination (Figure 6). According to the findings reported by Gowda et al., the use of appropriately tailored educational tools (websites) can change the attitudes of parents concerned about vaccine-induced autism [26]. It should be strongly emphasized that an association of vaccines, particularly MMR vaccines, with autism has repeatedly been disproved in numerous studies [27,28,29]. To talk with patients about preventive vaccinations, physicians should know the newest data available in literature. In order to avoid conflicting opinions in the health care professional community, information on this subject should be included in the medical curriculum and postgraduate courses. Moreover, the importance of medical education in the field of vaccinology is stressed by Marotta et al., who conclude that vaccine-associated knowledge is pivotal for shaping attitudes toward vaccinations in the total population [30]. Reduced vaccination rates, decrease herd immunity, resulting in higher incidence rates of infectious diseases, e.g., measles. According to the Brazilian study, the level of knowledge of students on this subject is insufficient [31].

### 4.2. Influenza Vaccinations

Influenza vaccinations are of special interest. Many countries have attempted to increase influenza vaccination coverage, particularly in risk groups and among health professionals. Unfortunately, our results confirm the pessimistic reports. The majority of young people, especially students (75.8%), are not vaccinated against influenza. Similar data are reported by G.Bonaccorsi et al.; 74.6% of health professionals have never been vaccinated against seasonal influenza, 13.1% once or twice in the last 3 seasons and 12.3% each year. Among students, the percentage of nonvaccinated individuals has been even higher, i.e., 79.7% (85% in our survey) [32] Given the issues of public health as well as high morbidity and mortality caused annually by the influenza virus, special attention should be paid and appropriate steps taken to encourage also young people who are not at high risk.

### 4.3. Knowledge on Vaccines and Their Public Perception

In our study, the respondents’ knowledge of vaccination was verified by asking about a disease that was eradicated through vaccination; 36.75% (95%CI: 35.53–37.97) of respondents gave the correct answers. Importantly, respondents who knew correct answers were much more likely to be supporters of vaccination, as compared to those who gave wrong answers *p* = 0.001 (Figure 4). It is surprising that during the period of an alarmingly increased incidence of measles cases and the widespread availability of information on this subject, 22.70% (95%CI: 21.64–23.76) of respondents in the study group considered measles to be eliminated.

To improve the public perception of vaccinations, clear and balanced media information on the safety and effectiveness of vaccination should be provided. In France, when new mandatory vaccination regulations were introduced (June 2017–May 2018), the media widely publicized the issue, yet the information was often contradictory. This resulted in a partial loss of confidence in vaccination and caused much controversy surrounding the new regulations [33]. The media can have significant effects on both positive and negative opinions regarding vaccination in the society [29] In our analysis, respondents with undecisive attitudes were statistically more likely to identify the media as their primary source of information (Figure 1), which has been confirmed by many authors demonstrating a significant role of the press, television and internet (in particular) in the recent antivaccination campaign [34,35,36,37,38].

Based on our findings demonstrating that the most important sources of information are physicians and media (including the internet), possible measures to combine these two forces should be considered. Margaret Stager has formulated some guidelines for physicians who want to share their knowledge through social media [39]. In the nearest future, this form of media-based information is believed to have particularly strong effects on public opinions. It is essential to disprove the vaccination-related myths and to highlight the risks associated with vaccine-preventable diseases. The appropriate data provided facilitate the parents’ vaccine decisions concerning themselves or their children, which in turn improves the health of the entire population [40].

As far as the measures to prevent vaccination refusals are concerned, the issue of making vaccinations mandatory through law is increasingly debated. According to K.Kieslich, the priority in the field of public health for the respective authorities and the society should be well-planned and organized education curricula regarding this form of prevention of infectious diseases, rather than further orders and measures of compulsion [41].

### 4.4. Local Situation in Poland

A survey carried out in Poland in 2015 concerning knowledge on influenza vaccinations revealed that the level of knowledge on this topic was insufficient. Effective education is needed to increase influenza vaccination rates. Similar to our study, the majority of respondents were women (72%) and young people (78% <35 years) [42]. In the second Polish study, the attitude of doctors toward preventive vaccinations was analyzed, 500 primary care physicians were interviewed. Older age of respondents, correctly addressing vaccination myths and use of one or more than one scientific sources of knowledge had a positive effect on attitudes toward vaccination. One of the conclusions of this study was a postulate to put more emphasis on the topic of vaccination in the process of the education of medical staff [43]. Our results also indicate that education is fundamental for shaping pro-vaccination attitudes. For several years now, social, state and local government actions have been carried out in Poland in order to popularize vaccination and build public confidence in this form of prophylaxis. Poland is a country with a long tradition of mandatory vaccinations, but not all vaccinations are reimbursed. Newer more expensive vaccines e.g., polyvalent preparations must be purchased by patients from their own funds. Such a situation is not favorable for the promotion of vaccinations, and experts for years have been postulating increased expenditure on the national vaccination program.

### 4.5. Study Limitations

The majority of respondents was a population of young adults, which could affect the external representativeness of our study population, but the statistical methods used allowed for obtaining the results presented and drawing conclusions. Moreover, our analysis did not include opinion on compulsory vaccination in the entire group of children and young people legally obliged to be vaccinated (0–19 years). Many aspects of vaccinations that may affect their perception were not analyzed in our survey study (e.g., costs to patients, fear of needles, concern about adverse reactions). This is due to the limited capacity of the questionnaire and the care taken to make it as brief and transparent as possible. One thought to bear in mind is that, as with most survey studies, the findings are based on self-reported data. Therefore, there may have been some misrepresentations caused e.g., by the respondents’ desire to present themselves in a more positive perspective. This study has a cross-sectional character, the findings can only be suggestive of causal relationships, such surveys are not able to test or confirm causal relationships.

## 5. Conclusions

Firstly, physicians and the medical community still exert decisive effects on attitudes toward vaccinations. Moreover, the quality of the information provided by them is of importance. Negative social attitudes toward vaccinations are most strongly determined by the conviction of an alleged relationship between vaccination and autism; therefore, skilful and competent provision of evidence-based information disproving this myth is essential.

### Practical Implications and Future Research

Physicians, scientists and representatives of health-promoting movements should be increasingly involved in media activities (internet, radio, television, press) and social fora to disprove the disinformation about vaccines. Furthermore, in order to mold correct attitudes of society toward vaccination, the substantive preparation of secondary school pupils, medical students, physicians and other healthcare professionals is essential for provision of coherent and uniform data as well as arguments demonstrating the benefits of mass vaccination.

The present study analyzed the material concerning the entire study population; further separate reports are planned to analyze and discuss the attitudes toward preventive vaccinations among students, health professionals and parents.

## Figures and Tables

**Table 1:** Questionnaire.

No.	Question	Answers
***B1***	Please indicate one source of information about vaccinations that you find most convincing	(1) medical sources (doctor, nurse, pharmacist) (2) media (press, radio, television, Internet) (3) environment (family, friends)
***B2***	Did you obtain information on vaccination from healthcare professionals during visits to primary care physicians (PCP) or specialist outpatient clinic?	(1) Yes, (2) No
***B3***	Using the school grade system, assess the level of vaccination information provided by health professionals	(1 worst–6 best)
***B4***	What was the most important source of negative opinions about vaccinations?	(1) medical sources, (2) media, (3) environment
***B6***	What opinion about vaccination was provided by health professionals?	(1) fully positive, (2) questioning the validity of certain vaccinations, (3) strongly negating the purposefulness of vaccinations
***B7***	Which of the infectious diseases has been completely eliminated by vaccination?”	(1) measles, (2) rabies, (3) smallpox, (4) whooping cough, (5) hepatitis C
***B8***	Do you consider yourself an advocate or opponent of vaccination?	(1) advocate, (2) opponent
***B9***	The statement that “vaccines cause autism” is:”	(1) true, (2) false
***B10***	In your opinion, should one be given mandatory, mandatory and recommended vaccines or not be given any vaccinations?	(1) mandatory, (2) mandatory and recommended vaccines, (3) no vaccinations
***B11***	Have you been vaccinated in the last three years?	(1) Yes, (2) No
***B12***	Are you vaccinated against seasonal influenza?	(1) Yes, (2) No
***B13***	What do you consider when choosing additional prophylaxis for yourself or your child?	(1) I want to immunize myself/child against all possible diseases, (2) I want to immunize myself/child against serious life-threatening diseases, (3) I want to vaccinate myself/child with advertised (“fashionable”) vaccines, (4) I would like to vaccinate myself/child with additional vaccines but this is beyond my budget, (5) I don’t think additional vaccinations are necessary, (6) I think vaccinations are harmful.
***B14***	If you consider vaccination to be harmful, which of the following diseases is in your opinion caused by vaccination:	(1) allergy, (2) neurological disorders, (3) autism, (4) diabetes, (5) immunosuppression, (6) myocarditis, (7) sudden infant death, (8) all of the above-mentioned diseases.

**Table 2:** Study group characteristics.

Determinants		Number (*n*)	Percentage (%)	t-Statistics (*p* Value)
Respondents in general		6432	100	
Gender	Female	4303	66.9	93.25 (0.000)
	Male	2129	33.1	93.26 (0.000)
Age	18–25	4995	77.68	32.46 (0.000)
	26–35	697	10.06	131.85 (0.000)
	36–45	302	4.7	3.85 (0.000)
	>45	488	7.58	6.326 (0.000)
Education	Elementary	1031	16.1	14.07 (0.000)
	Secondary	272	4.2	3.45 (0.000)
	College students	4048	62.9	82.84 (0.000)
	Higher	1081	16.8	14.77 (0.000)
Financial status	Bad	97	1.5	1.22 (0.112)
	Medium	3039	47.2	52.12 (0.000)
	Good	3296	51.2	58.81 (0.000)
Having children	Yes	1038	16.1	14.11 (0.000)

**Table 3:** Logistic regression model parameters and odds ratios-dependent variable: answer to the question B8: “Do you consider yourself an advocate/opponent of vaccination?” (advocate (1), opponent (2)). Bold values denote statistical significance at the *p* < 0.05 level.

Variable	β	SE β	Wald’s χ^2^	e^β^ OR (95% CI)	*p*
Constant	1.16	0.80	2.09	NA	0.14
Sex (1-female, 2-male)	0.08	0.12	0.49	1.09 (0.86–1.36)	0.48
**Age**	**−0.43**	**0.11**	**16.29**	**0.28** **(0.53–0.80)**	**<0.001**
Level of education (elementary (1)–higher education (4))	0.14	0.08	2.87	1.53 (0.98–1.36)	0.09
Financial situation (bad (1)–good (3))	−0.06	0.11	0.27	0.90 (0.77–1.16)	0.60
Children (yes (1), no (2))	−0.09	0.23	0.14	0.92 (0.59–1.43)	0.71
**Fact of obtaining information on vaccinations from health care professionals [B2]** **(1- yes, 2- no)**	**0.69**	**0.12**	**33.41**	**2.00** **(1.58–2.53)**	**<0.001**
Level of information on vaccination received from health professionals [B3] (worst (1)–best (6))	−0.01	0.05	0.03	0.96 (0.9–1.09)	0.87
**Opinion on vaccination that you obtained from health care providers [B6]** **(positive (1)–fully negative (3))**	**0.65**	**0.10**	**44.41**	**3.67** **(1.58–2.32)**	**<0.001**
**“Which of the infectious diseases has been eliminated thanks to vaccinations?” [B7]** **wrong answer (0), correct answer (1)**	**−0.80**	**0.11**	**45.44**	**0.45** **(0.09–0.18)**	**<0.001**
**“Vaccines cause autism” [B9]** **true (1), false (2)**	**−2.06**	**0.19**	**113.37**	**0.13** **(0.36–0.57)**	**<0.001**
**For the entire model: χ^2^ = 344.18 *p* = 0.0000**					

**Table 4:** Logistic regression model parameters and odds ratios-dependent variable: answer to the question B12 “Are you vaccinated against seasonal influenza?” (1-YES, 2-NO). Bold values denote statistical significance at the *p* < 0.05 level.

Variable	β	SE β	Wald’s χ^2^	e^β^ OR (95% CI)	*p*
Constant	0.31	0.59	0.27	NA	0.60
Sex (1-female, 2-male)	−0.12	0.09	1.69	0.89 (0.74–1.06)	0.19
**Age**	**−0.40**	**0.06**	**42.76**	**0.30** **(0.59–0.76)**	**<0.001**
**Level of education** **(elementary (1)–higher education (4))**	**0.21**	**0.05**	**15.14**	**1.85** **(1.1–1.36)**	**<0.001**
Financial situation (bad (1)–good (3))	0.00	0.02	0.01	1.00 (0.96–1.03)	0.93
**Children** **(yes (1), no (2))**	**0.42**	**0.14**	**8.39**	**1.51** **(1.14–2.01)**	**0.004**
**Fact of obtaining information on vaccinations from health care professionals [B2]** **(yes (1), no (2))**	**0.44**	**0.10**	**19.91**	**1.55** **(1.28–1.88)**	**<0.001**
**Level of information on vaccination received from health professionals [B3]** **(worst (1)–best (6))**	**0.07**	**0.03**	**4.59**	**1.41** **(1.01–1.14)**	**0.032**
Opinion on vaccination that you obtained from health care providers [B6] (positive (1)–fully negative (3))	0.12	0.08	2.31	1.27 (0.97–1.31)	0.13
**“Which of the infectious diseases has been eliminated thanks to vaccinations?” [B7]** **wrong answer (0), correct answer (1)**	**−0.39**	**0.08**	**20.86**	**0.68** **(0.58–0.80)**	**<0.001**
**“Vaccines cause autism” [B9]** **true (1), false (2)**	**−0.39**	**0.19**	**4.14**	**0.68** **(0.47–0.99)**	**0.04**
**For the entire model: χ^2^ = 270.86 *p* = 0.0000**					

## References

[1] Centers for Disease Control and Prevention Ten Great Public Health Achievements—United States, 1900–1999. https://www.cdc.gov/mmwr/preview/mmwrhtml/00056796.htm.

[2] Kempe A., Daley M.F., McCauley M.M., Crane L.A., Suh C.A., Kennedy A.M., Basket M.M., Stokley S.K., Dong F., Babbel C.I. (2011). Prevalence of Parental Concerns About Childhood Vaccines. Am. J. Prev. Med..

[3] Jacobson R.M., St. Sauver J.L., Finney Rutten L.J. (2015). Vaccine hesitancy. Mayo Clin. Proc..

[4] Kestenbaum L.A., Feemster K.A. (2015). Identifying and addressing vaccine hesitancy. Pediatr. Ann..

[5] Lane S., MacDonald N.E., Marti M., Dumolard L. (2018). Vaccine hesitancy around the globe: Analysis of three years of WHO/UNICEF Joint Reporting Form data-2015–2017. Vaccine.

[6] Charron J., Gautier A., Jestin C. (2020). Influence of information sources on vaccine hesitancy and practices. Médecine Mal. Infect..

[7] Gandhi C.K., Patel J., Zhan X. (2020). Trend of influenza vaccine Facebook posts in last 4 years: A content analysis. Am. J. Infect. Control.

[8] Boyle J., Berman L., Nowak G.J., Iachan R., Middleton D., Deng Y. (2020). An assessment of parents’ childhood immunization beliefs, intentions, and behaviors using a smartphone panel. Vaccine.

[9] MacDonald N.E. (2015). Vaccine hesitancy: Definition, scope and determinants. Vaccine.

[10] Dubé E., Gagnon D., Nickels E., Jeram S., Schuster M. (2014). Mapping vaccine hesitancy-Country-specific characteristics of a global phenomenon. Vaccine.

[11] Dubé E., Gagnon D., MacDonald N.E., Eskola J., Liang X., Chaudhuri M., Dube E., Gellin B., Goldstein S., Larson H. (2015). Strategies intended to address vaccine hesitancy: Review of published reviews. Vaccine.

[12] Smith T.C. (2017). Vaccine Rejection and Hesitancy: A Review and Call to Action. Open Forum Infect. Dis..

[13] Dubé E., Bettinger J., Fisher W., Naus M., Mahmud S., Hilderman T. (2016). Vaccine acceptance, hesitancy and refusal in Canada: Challenges and potential approaches. Can. Commun. Dis. Rep..

[14] Nowicka P.M., Izdebski R., Kitowska W., Janiec J., Radziszewski F., Henszel Ł. (2019). Review of measles-related events recorded by the National IHR Focal Point in Poland in the years 2016–2018. Przegl. Epidemiol..

[15] Arede M., Bravo-Araya M., Bouchard É., Gill G.S., Plajer V., Shehraj A., Shuaib Y.A. (2019). Combating vaccine hesitancy: Teaching the next generation to navigate through the post truth era. Front. Public Heal..

[16] Kowalska M., Gajda M., Barański K., Braczkowska B. (2019). Sources of parental knowledge about the safety of vaccinations in Poland. Health Promot. Int..

[17] Augustynowicz A., Wrześniewska-Wal I. (2013). Legal aspects of compulsory immunization of children. Pediatr. Pol..

[18] Główny Inspektor Sanitarny do RPO: 43 tys. osób bez obowiązkowych szczepień. https://www.rpo.gov.pl/pl/content/GIS-do-RPO-43-tys.-osob-odmawia-obowiazkowych-szczepien.

[19] CBOS (Centrum Badania Opinii Społecznej) Polacy o obowiązku szczepienia dzieci. https://www.cbos.pl/SPISKOM.POL/2017/K_100_17.PDF.

[20] Facciolà A., Visalli G., Orlando A., Bertuccio M.P., Spataro P., Squeri R., Picerno I., Di Pietro A. (2019). Vaccine hesitancy: An overview on parents’ opinions about vaccination and possible reasons of vaccine refusal. J. Public health Res..

[21] Gust D., Woodruff R., Kennedy A., Brown C., Sheedy K., Hibbs B. (2003). Parental perceptions surrounding risks and benefits of immunization. Semin. Pediatr. Infect. Dis..

[22] Shui I.M., Weintraub E.S., Gust D.A. (2006). Parents Concerned About Vaccine Safety. Am. J. Prev. Med..

[23] Kennedy A.M., Brown C.J., Gust D.A. (2005). Vaccine beliefs of parents who oppose compulsory vaccination. Public Health Rep..

[24] Shen S.C., Dubey V. (2019). Addressing vaccine hesitancy: Clinical guidance for primary care physicians working with parents. Can. Fam. Phys..

[25] Berry N.J., Danchin M., Trevena L., Witteman H.O., Kinnersley P., Snelling T., Robinson P., Leask J. (2018). Sharing knowledge about immunisation (SKAI): An exploration of parents’ communication needs to inform development of a clinical communication support intervention. Vaccine.

[26] Gowda C., Schaffer S.E., Kopec K., Markel A., Dempsey A.F. (2013). A pilot study on the effects of individually tailored education for MMR vaccine-hesitant parents on MMR vaccination intention. Hum. Vaccin. Immunother..

[27] Hviid A., Hansen J.V., Frisch M., Melbye M. (2019). Measles, Mumps, Rubella Vaccination and Autism. Ann. Intern. Med..

[28] Smeeth L., Cook C., Fombonne E., Heavey L., Rodrigues L.C., Smith P.G., Hall A.J. (2004). MMR vaccination and pervasive developmental disorders: A case-control study. Lancet.

[29] Taylor L.E., Swerdfeger A.L., Eslick G.D. (2014). Vaccines are not associated with autism: An evidence-based meta-analysis of case-control and cohort studies. Vaccine.

[30] Marotta C., Raia D.D., Ventura G., Casuccio N., Dieli F., D’Angelo C., Restivo V., Costantino C., Vitale F., Casuccio A. (2017). Improvement in vaccination knowledge among health students following an integrated extra curricular intervention, an explorative study in the University of Palermo. J. Prev. Med. Hyg..

[31] Mizuta A.H., De Menezes Succi G., Montalli V.A.M., De Menezes Succi R.C. (2019). Perceptions on the importance of vaccination and vaccine refusal in a medical school. Rev. Paul. Pediatr..

[32] Bonaccorsi G., Santomauro F., Porchia B.R., Niccolai G., Pellegrino E., Bonanni P., Lorini C. (2015). Beliefs and opinions of health care workers and students regarding influenza and influenza vaccination in Tuscany, Central Italy. Vaccines.

[33] Mitilian E., Malli F., Verger P. (2020). Image of the new vaccination obligation through the media. Vaccine.

[34] Donzelli G., Palomba G., Federigi I., Aquino F., Cioni L., Verani M., Carducci A., Lopalco P. (2018). Misinformation on vaccination: A quantitative analysis of YouTube videos. Hum. Vaccines Immunother..

[35] Hussain H., Omer S.B., Manganello J.A., Kromm E.E., Carter T.C., Kan L., Stokley S., Halsey N.A., Salmon D.A. (2011). Immunization safety in US print media, 1995–2005. Pediatrics.

[36] Kennedy A., LaVail K., Nowak G., Basket M., Landry S. (2011). Confidence About Vaccines in The United States: Understanding Parents’ Perceptions. Health Aff..

[37] Davies P., Chapman S., Leask J. (2002). Antivaccination activists on the world wide web. Arch. Dis. Child..

[38] Gowda C., Dempsey A.F. (2013). The rise (and fall?) of parental vaccine hesitancy. Hum. Vaccines Immunother..

[39] Stager M. News & Views: Using Social Media as a Healthcare Provider—What Role Do You Play?. https://www.chop.edu/news/news-views-using-social-media-healthcare-provider-what-role-do-you-play.

[40] Vivion M., Hennequin C., Verger P., Dubé E. (2020). Supporting informed decision-making about vaccination: An analysis of two official websites. Public Health.

[41] Kieslich K. (2018). Addressing vaccination hesitancy in Europe: A case study in state–society relations. Eur. J. Public Health.

[42] Kuchar E., Ludwikowska K., Szenborn L., Antczak A., Mastalerz–Migas A., Nitsch–Osuch A. (2018). Knowledge Regarding Influenza and Influenza Vaccination in General Population: Results of a National Survey in Poland. Adv. Exp. Med. Biol..

[43] Stefanoff P., Sobierajski T., Bulinska-Stangrecka H., Augustynowicz E. (2020). Exploring factors improving support for vaccinations among Polish primary care physicians. PLoS ONE.

